# Mefloquine safety and tolerability in pregnancy: a systematic literature review

**DOI:** 10.1186/1475-2875-13-75

**Published:** 2014-02-28

**Authors:** Raquel González, Urban Hellgren, Brian Greenwood, Clara Menéndez

**Affiliations:** 1 Barcelona Centre for International Heath Research (CRESIB, Hospital Clínic- Universitat de Barcelona), Rosselló 132, 4-2, Barcelona E-08036, Spain; 2 Manhiça Health Research Centre (CISM), Maputo, Mozambique; 3 Unit of Infectious Diseases, Department of Medicine, Karolinska University Hospital Huddinge, Karolinska Institutet, Stockholm, Sweden; 4 Faculty of Infectious and Tropical Diseases, London School of Hygiene & Tropical Medicine, London, UK

**Keywords:** Mefloquine, Malaria, Pregnancy, Safety, Intermittent preventive treatment

## Abstract

**Background:**

Control of malaria in pregnant women is still a major challenge as it constitutes an important cause of maternal and neonatal mortality. Mefloquine (MQ) has been used for malaria chemoprophylaxis in non-immune travellers for several decades and it constitutes a potential candidate for intermittent preventive treatment in pregnant women (IPTp).

**Methods:**

The safety of MQ, including its safety in pregnancy, is controversial and a continuing subject of debate. Published studies which evaluated the use of MQ for malaria prevention or treatment in pregnant women and which reported data on drug tolerability and/or pregnancy outcomes have been reviewed systematically.

**Results:**

Eighteen articles fitted the inclusion criteria, only one study was double-blind and placebo controlled. No differences were found in the risk of adverse pregnancy outcomes in women exposed to MQ compared to those exposed to other anti-malarials or to the general population. MQ combined with artesunate seems to be better tolerated than standard quinine therapy for treatment of non-severe falciparum malaria, but a MQ loading dose (10 mg/kg) is associated with more dizziness compared with placebo. When used for IPTp, MQ (15 mg/kg) may have more side effects than sulphadoxine- pyrimethamine.

**Conclusions:**

In the published literature there are no indications that MQ use during pregnancy carries an increased risk for the foetus. Ideally, the use of MQ to prevent malaria should be based on a risk-benefit analysis of adverse effects against the risk of acquiring the infection. For this purpose double-blinded randomized controlled trials in African pregnant women are much needed.

## Background

Malaria in pregnancy continues to be a global health problem, accounting for 15% of maternal deaths in some malaria endemic regions [1,2]. It also contributes to low birth weight (LBW) (<2,500 g), either through intrauterine growth retardation or pre-term delivery and to the occurrence of severe anaemia in the mother [3-15]. The World Health Organization (WHO) recommends a package of interventions to prevent the consequences of malaria during pregnancy in areas with stable transmission in sub-Saharan Africa including the use of insecticide-treated nets (ITNs), intermittent preventive treatment (IPT) with sulphadoxine-pyrimethamine (SP) and effective case management of malaria and anaemia [16]. In October 2012, the WHO policy recommendation on IPT in pregnancy (ITPp) with SP was updated and IPTp with SP is now recommended at each scheduled antenatal care (ANC) visit for pregnant women living in areas of moderate-to-high malaria transmission, provided that each dose is separated by at least a month [17]. The emergence of *Plasmodium falciparum* parasites resistant to SP has raised concern over the long-term efficacy and effectiveness of SP as IPTp [18-21]. As a result, alternative drugs are being evaluated for IPTp to replace SP in the short or the long term.

Mefloquine (MQ) has many of the characteristics needed for an anti-malarial to replace SP for IPTp [22]. These include: 1) a long half-life (median between 14 and 28 days at curative doses and between 12 and 17 days at prophylactic doses); 2) single dose administration; 3) a well-characterized pharmacokinetic profile in pregnant women [23-25]; 4) infrequent MQ resistance in Africa; and, 5) an acceptable reproductive toxicity profile in animal studies. As a consequence, MQ is considered appropriate for chemoprophylaxis for pregnant women travellers of all gestational ages to high risk areas by various expert agencies such as the United States (US) Centers for Disease Control and Prevention (CDC) and the French Reference Centre on Teratogenic Agents (CRAT) [26,27]. In addition, the drug was recently reclassified as pregnancy category B (though initially rated as C) by the US- Food and Drug Administration (FDA) [28].

MQ was developed by the US Army in the late 1970s. It belongs to the arylaminoalcohols group of anti-malarial drugs and has blood schizonticidal properties [29]. The most common adverse effects related to its use are gastrointestinal and neurological. Severe central nervous system side effects occur in about 1:10,000 travellers taking MQ as chemoprophylaxis [30]. Risk factors reported to be associated with MQ-induced neuropsychiatric adverse events include a previous history of psychiatric problems, female sex, low body mass index (BMI) and first-time use of the drug [31]. Mefloquine is contra-indicated in subjects with a history of a neuropsychiatric illness, including epilepsy. The incidence of adverse events is higher when MQ is administered at the recommended treatment dosage (25 milligram (mg)/ kilogram (kg)) as compared to lower doses (15 mg/kg), suggesting a dose-related effect [32]. The frequency of adverse events is considerably lower when the drug is used at prophylactic doses (250 mg/week) than when it is used for treatment. Recently, the FDA has released a safety communication raising concerns about possible long-term psychiatric and neurological side effects following MQ use [33]. The drug is currently one of the most controversial anti-malarial medicines and the target of various special interest groups following its massive administration to US troops deployed in endemic areas since 1992 [34].

In view of the potential use of MQ for IPTp in malaria-endemic areas and existing concerns regarding its safety, a systematic literature review on the safety of MQ when given in pregnancy was carried out. The study included published articles reporting data on the safety of MQ administered for malaria treatment or prevention in pregnant women. This review also provided a background paper for WHO’s Technical Expert Group on IPTp, which met in Geneva in July 2013 to consider the potential use of MQ for IPTp.

## Methods

A comprehensive search for data on the safety of MQ in pregnancy in medical databases (PubMed, the Cochrane library, US National Institutes of Health Clinical Trials data base [35], WHO library) was made, and non-medical search engines were interrogated using “mefloquine”, “pregnancy” as keywords/search terms from April to June 2013. Thirty-five abstracts were selected from the 136 initially listed titles for further review. Special consideration was given to original articles and systematic reviews but reports of case series were also included. An additional search introducing “safety” as a search term resulted in a further 25 articles for screening. Additional references were obtained from references provided in the articles identified through the search. The current review focuses on the safety of MQ use in pregnant women in terms of pregnancy outcomes and drug tolerability. Criteria for inclusion in the review were published articles written in English reporting results of studies that evaluated the safety of MQ (used alone or combined with other anti-malarials) in pregnant women for treatment and/or prevention of malaria. The main findings and conclusions generated by all the studies reviewed are organized by topic and the key parameters of these studies are summarized in two tables.

## Results

**Figure 1:**
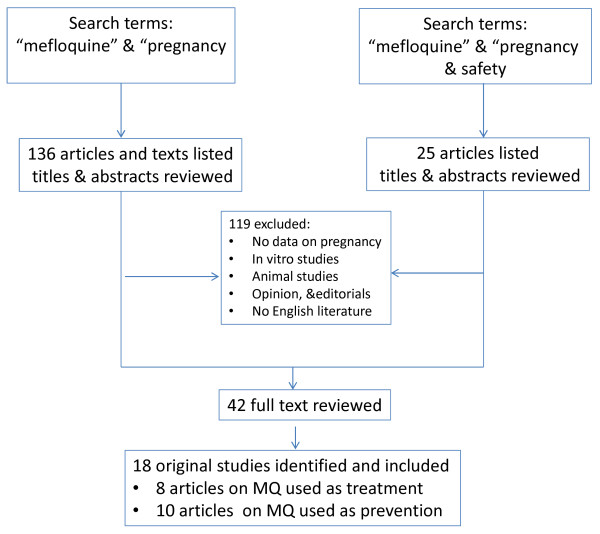
Diagram flow of articles selected.

Figure 1 shows the flow diagram of the article selection process. Eighteen articles which met the inclusion criteria were included in the final selection: eight reported safety data of MQ when used for malaria treatment (Table 1) and ten evaluated MQ in pregnant women for malaria prevention (See Additional file 1).

**Table 1:** Studies which evaluated the safety of mefloquine for treatment of malaria in pregnant women

**Reference**	**Study year and location**	**Study design**	**Study women**	**MQ safety on pregnancy outcomes**	**MQ tolerability**	**Comments**
Harinasuta *et al.* 1990 [45]	Thailand	Clinical trial which compared MQ to QN for the treatment of multi-resistant falciparum malaria	N = 85 women (all trimesters) treated with MQ *vs* N = 72 treated with QN	No differences in stillbirth rates between groups	All mild and transient adverse events.	Small sample size
						Limited information on procedures and results available
Okeyeh *et al.* 1996 [47]	Nigeria	Non comparative MQ treatment study in pregnant women (12.5 mg/kg)	N = 33 women in 2^nd^ and 3^rd^ trimester	No stillbirths and congenital malformations reported	Minimal side effects	Small sample size
						Low dose of MQ used
Sowunmi *et al.* 1998 [49]	Nigeria	Open label trial which compared artemether to artemether + MQ in the treatment of uncomplicated malaria	N = 45 women in 2^nd^ and 3^rd^ trimesters	No abortion, stillbirth or congenital anomalies were observed	Minimal adverse events reported in the artemether – MQ group (dizziness and abdominal pain) in 2/45 patients	Small sample size
			n = 23 artemether			Open label trial
			n = 22 artemether + MQ			
McGready *et al*. 1998 [43]	1991-96	Non- randomized comparative MQ treatment study, cohort series	N = 372	Similar rates of congenital anomalies and stillbirths among groups	The most common adverse effects following MQ were dizziness (36%) and anorexia (23%)	Open label cohort series Groups not well matched
	Thailand		n = 194 treated with MQ (in 2^nd^ and 3^rd^ trimesters)			
			n = 93 treated with QN			
			n = 85 MQ + QN			
Nosten *et al*. 1999 [44]	1991-94	Retrospective analysis of the pregnancy outcomes of women exposed to MQ compared to those not exposed (based on ANC registries and self-reported information from interviews)	N = 208 pregnancies exposed to MQ (mainly 2^nd^ and 3^rd^ trimesters) *vs*	Increased risk of reported stillbirths in women exposed to MQ:	No data available	Analysis with several limitations
	Thailand		N = 656 exposed to QN *vs*	(9/208) 4.5% (MQ group) *vs*		1) Four women out of the nine with a stillbirth had been exposed to other anti-malarials;
			N = 909 exposed to other anti-malarials *vs*	(10/656) 1.6% (QN group) *vs*		
			N = 2,470 not exposed to anti-malarials	(12/909) 1.4% (other anti-malarials) *vs*		
				(40/2470) 1.8% (not exposed)		2) Recall bias possible (results based on self-reported data)
McGready *et al.* 2000 [40]	1995-97	Open randomized comparison of different malaria treatments in pregnant women in the 2^nd^ and 3^rd^ trimesters	N = 108	No differences in the rates of congenital anomalies, stillbirths or birth weight between the treatment groups	No serious adverse effects were reported	Small sample size
	Thailand		n = 42 QN 7 days		Dizziness was more frequent in the QN group than in the MQ (87 *vs* 45%)	MQ combined with AS
			*vs*			Open label
			n = 66 MQ (25 mg/kg) + AS 3 days			
Bounyasong 2001 [48]	Thailand	Open randomized comparison of different malaria treatments in pregnant women in the 2^nd^ and 3^rd^ trimesters	N = 60	No data available	QN group reported more adverse effects than the MQ group (nausea, vomiting, vertigo, tinnitus and hypoglycaemia)	Small sample size
			n = 29 QN 7 days *vs*			MQ combined with AS
			n = 28 AS + MQ			Open label
			3 Lost to follow-up			
Adam *et al.* 2004 [46]	1998-2001	Prospective study which evaluated the efficacy and safety of MQ in women who presented with malaria after a full course of CQ therapy	N = 40	No abortion, stillbirth and congenital anomalies were observed	35% reported nausea and 17.5% itching	Small sample size
	Sudan		Pregnant women in the 2^nd^ or 3^rd^ trimester of gestation			Non comparative study

### The safety of mefloquine when used for the treatment of malaria in pregnancy

The first reports on the use of MQ in pregnant women are from the late 1980s [36,37]. Most experience on its use in treatment of malaria in pregnancy comes from Southeast Asia where MQ was administered primarily in combination with artesunate (AS) and where increasing resistance to MQ has been reported [38,39]. In Thailand, this combination (MQ-AS) was shown to be more effective than quinine (QN) in clearing parasites and fever in pregnant women with uncomplicated malaria [40,41]. Studies are currently on going in sub-Saharan Africa to evaluate MQ-AS efficacy for malaria treatment in pregnancy [42].

A non-randomized, comparative MQ treatment study conducted in 372 pregnant women with uncomplicated falciparum malaria in Thailand between 1991 and 1996 reported no differences in the rates of congenital anomalies and stillbirth among women who were treated with a single dose of MQ (25 mg/kg; n = 194) and those treated with QN for seven days (30 mg/kg/day; n = 93), or MQ + QN (n = 85) [43]. The most common adverse effects following MQ administration were dizziness (36%) and anorexia (23%) and the most common adverse effects after QN were dizziness (42%) and tinnitus (35%). The study was not blinded and the groups were not well matched and thus findings on tolerability are of limited value.

In 1999, a report was published on a retrospective analysis of the pregnancy outcomes of women exposed to MQ in Thailand, based on ANC registries and self-reported information from interviews [44]. This study showed an increased risk of stillbirths in women treated with 25 mg/kg of MQ (9/208, 4.5%) compared to those treated with other anti-malarials (10/656 [1.6%] in those exposed to QN, and 12/909 [1.4%] in those exposed to other anti-malarials). Despite the fact that the study had some limitations (such as possible recall bias and the small number of stillbirths observed in the MQ group), the article led CDC to recommend that MQ should not be used for malaria treatment in pregnancy if other effective anti-malarial medicines were available, as well as raising much general concern and open debate about the safety of this drug in pregnancy. Harinasuta *et al.* had previously reported no differences in the rates of stillbirths between study groups in a small clinical trial which had compared MQ (two doses of 500 mg, n = 85) with QN (1800 mg for seven days, n =72) for the treatment of multiresistant falciparum malaria in pregnant Thai women [45]. Another randomized study from the same region in Thailand, which compared MQ-AS (n = 54) with QN (n = 42) for the treatment of falciparum malaria in pregnant women in the second and third trimesters, found no differences in the rates of congenital anomalies, stillbirths or birth weight between the treatment groups [40]. Those treated with QN (10 mg/kg × 3 for seven days) compared with those treated with MQ (15 mg/kg on day 1 + 10 mg/kg on day 2) and AS (4 mg/kg/day for three days) had significantly more dizziness (87 *versus* 45%) and tinnitus (66 *versus* 17%). However, the study was not blinded and included only a small number of women.

A small, non-comparative study (n = 40) conducted between 1998 and 2001 among Sudanese pregnant women who presented with malaria after a full course of chloroquine (CQ) therapy and who were then treated with MQ (25 mg/kg), concluded that MQ could be used safely during the second and third trimesters of gestation as no abortions, stillbirths or congenital anomalies were observed [46]. The main complaints of recipients were nausea and itching. In a similar, non-comparative study undertaken in Nigeria, where a much lower MQ dose (12.5 mg/kg) was used, only minimal side effects were reported [47]. Two additional, small, randomized but not blinded *P. falciparum* treatment studies have been published [48,49]. In a study from Nigeria (n = 45), intramuscular artemether for five days was compared with artemether given in combination with MQ 15 mg/kg on the first two days of treatment. Reported adverse effects were minimal and all newborn babies were normal at birth [49]. The second study from Thailand (n = 60) found that women treated with standard QN regimen (10 mg/kg/day for seven days) in the second to third trimester had significantly more nausea, vomiting, vertigo tinnitus and hypoglycaemia compared with those who received the standard MQ (25 mg/kg) + AS regimen [48]. The physical and neurological developments of the babies in this study were normal (followed up to 12 months after delivery) and there were no congenital abnormalities.

### The safety of mefloquine when used for the prevention of malaria in pregnancy

One of the first reported studies on the use of MQ as prophylaxis in pregnancy described a placebo-controlled, double-blind trial conducted in 1987-90 in Thailand [50]. This trial, which evaluated MQ efficacy (250 mg/kg weekly) as malaria prophylaxis in pregnancy, enrolled 339 women in the second trimester of gestation in two study phases. The first phase was conducted between 1987 and 1988 and enrolled 60 women in the MQ group (they received an initial loading dose of 10 mg/kg before starting weekly prophylaxis) and 59 in the placebo group. The subsequent, second phase was conducted between 1989 and 1990 and enrolled 111 in the MQ group and 109 in the placebo arm. The MQ prophylactic dose used was the standard 250 mg once weekly for the first four weeks after which it was reduced to 125 mg once weekly until delivery. Overall, the rates of abortions, congenital anomalies, prematurity, and stillbirths were similar between groups. An increased risk of stillbirth was observed in the MQ group (12.5 *versus* 0%) in the first phase of the trial but this was not confirmed in the second phase. During the same period, pregnant women attending the same ANC but not participating in the study had a 6.7% rate of stillbirth. In the second phase, a weekly questionnaire, which asked about 20 symptoms, was used. There were no differences between the placebo and the MQ groups in reported adverse events. In addition, there were no differences in terms of liver, renal, neurological, or cardiac toxicity and no serious drug-related side effects were reported. The only significant finding was that MQ loading caused more transient dizziness than the placebo (28 *versus* 14%). The study concluded that MQ was safe and effective in the second half of pregnancy and that MQ prophylaxis was well tolerated.

During the same period, the Mangochi Malaria Research Project compared four malaria preventive regimens in pregnant Malawian women: 1) CQ treatment in an initial dose of 25 mg/kg followed by 300 mg weekly (n = 741); 2) CQ 25 mg/kg monthly (n = 1,459); 3) CQ 300 mg weekly (n = 661); and, 4) MQ in an initial treatment dose of 750 mg followed by 250 mg weekly (n = 932) [14,51,52]. This large, open-label trial enrolled 4,187 women and found similar overall rates of reported adverse effects following each treatment, as well as similar rates of abortion and stillbirth between groups. However, women who received MQ reported less itching and more dizziness compared to those who received CQ, although this was a non-blinded study and significance testing was not performed. The frequency of reported adverse events was lower after the fourth dose than after the first dose.

A subsequent analysis of a case series of 72 American soldiers who took weekly MQ prophylaxis without prior knowledge of their pregnancy showed a high frequency of spontaneous abortion (12/72) [53]. However, the authors considered that the high number of reported elective abortions (n = 17), losses to follow-up (n = 19) and potential exposure to other stress factors could have increased the rate of abortions in this particular population. In addition, no control group was available for comparison.

In 1998, a study of 1,627 reports of MQ exposure during pregnancy received by the Roche post-marketing surveillance system (mainly for chemoprophylaxis) between 1986 and 1996, reported a 4% prevalence of congenital malformations in infants of women in the cohort, a prevalence similar to that found in the general population [54]. The study included over 600 reports on MQ exposure during the 1^st^ trimester of gestation and it concluded that MQ could be used in pregnant women for prophylaxis. Phillips-Howard *et al.* also found no difference in the rates of adverse pregnancy outcomes among women exposed to MQ (n = 99) compared to women exposed to other anti-malarials (n = 137) in an analysis of reported use of MQ during the first trimester of pregnancy in European travellers [55].

More recently, an open-label, randomized, controlled trial (RCT) which compared MQ (15 mg/kg) with SP for IPTp was conducted in HIV-negative, pregnant women (805 in the MQ group and 804 in the SP) in Benin from 2005 to 2008 [22]. In this study, the incidence of spontaneous abortions (0.4% in MQ and 0.1% in the SP group), stillbirths (2.8% in the MQ and 2% in the SP group) and congenital anomalies (1% in the MQ and 0.5% in the SP group) did not differ significantly between groups. On the other hand, based on a questionnaire collected one week after drug intake, women who received MQ had a much higher frequency of reported adverse events than those who received SP: 52% vomiting and 50% dizziness, compared to 12 and 13% in the SP group, respectively. Most of the symptoms were mild and resolved quickly and spontaneously. The study was not blinded, which makes tolerability assessment difficult. The authors also suggest a possible enhanced anticipation of adverse events in the community where discussions had been organized before the first administration of IPTp.

A later analysis, which used data from the Beninese RCT described above and from another IPTp-MQ open trial, which compared MQ tolerability between HIV-infected (n = 94) and uninfected pregnant women (n = 385), found that adverse events such as vomiting and dizziness were less frequently reported in HIV-infected women than in uninfected women (33 *versus* 56% and 39 *versus* 51%, respectively) [56]. In both studies, adverse events were more frequent after the first IPTp-MQ than at the second. However, this is a comparison between two different, non-blinded studies using different protocols and done during different time periods so validity of this comparison must be interpreted with great caution.

A further analysis of 2,506 reports of MQ exposure during pregnancy, mainly when used for chemoprophylaxis, from the F Hoffman-La Roche global drug safety database has recently been published and concluded that the prevalence of birth defects (4.4%) (43/978) and foetal loss were comparable in women exposed to MQ in pregnancy to background rates of the general population [57]. This analysis included part of the reports analyzed previously by Phillips-Howard *et al.*[55].

## Discussion

In spite of several decades of experience with the use of MQ in the treatment of malaria, reports of only two RCTs were found which specifically evaluated MQ safety in pregnant women and only one of these was blind and placebo controlled [22,50]. This may be due to the fact that pregnant women are systematically excluded from drug trials for ethical, legal and sociological concerns because of fear of toxicity to the foetus [29].

The evidence provided by one large but not randomized, nor blinded study suggests that the tolerability to MQ when used as prophylaxis in pregnant women is similar to that of CQ, although the risk of dizziness might be higher with MQ [51]. The only randomized, controlled, double-blind trial which compared MQ tolerability to placebo, did not find differences in the rates of reported adverse effects between study arms in those not given a MQ loading dose [50]. An interim WHO report on MQ tolerability in pregnancy (Urban Hellgren, unpublished) points out the need for blinded studies to accurately estimate common and subjective side effects, especially for evaluation of tolerability and side effects of disputed medicines such as MQ [34,58]. This is particularly the case when cases of serious adverse events are disseminated widely in the general media. This may have contributed to the considerable controversy among international experts regarding the tolerability of MQ prophylaxis *versus* alternative regimens in travellers [26]. In the IPTp randomized but not blinded trial conducted in Benin, a dose of 15 mg of MQ was poorly tolerated compared with SP, with higher frequencies of adverse events such as vomiting and dizziness [22]. However, when study participants are aware of the possibility of specific adverse events either through the consent form or through general knowledge of the drug, reporting rates of those adverse events typically increase [59]. Such knowledge is also likely to affect the evaluation of relatedness to the drug treatment by the investigator. In this trial, it was observed that the frequency of related adverse events decreased with increasing number of doses, as in other studies of chemoprophylaxis with MQ in pregnancy, but also in reports from travellers indicating that a true tolerance effect might play a role [26,51]. However, the incidence of adverse events reporting also decreases with time in the placebo group in absence of drug treatment [60,61].

Neuro-psychiatric adverse events (such as anxiety, depression, behavioral changes, etc.) are difficult to assess and monitor, especially in resource- constrained settings where malaria is endemic. Thus it is possible that such adverse events are underreported, which makes assessment of these adverse events challenging. In addition, malaria symptoms in pregnant women may be difficult to distinguish from adverse events and consequently relatedness to the drug may be particularly difficult to assess [62].

Only a few small studies were found to have assessed foetal safety of MQ when administered for malaria treatment in pregnant women. Most of the safety data on MQ use in pregnancy come from the post-marketing surveillance system of the manufacturer (dominated by exposure as chemoprophylaxis) and from retrospective studies or studies from Southeast Asia [54,57,63,64]. There is a relative lack of safety studies on MQ treatment in pregnant women in sub-Saharan Africa.

Concerns regarding a potential increased risk in the rates of adverse pregnancy outcomes in women who have received MQ during pregnancy constitute one of the main controversial issues regarding MQ safety. However, the results from the single study, which reported an association between MQ treatment and stillbirth in Thailand [44] which initiated these concerns, have not been confirmed in larger studies conducted in sub-Saharan Africa [14,22]. Most of the identified studies included few participants and were underpowered to appropriately assess the risk of adverse pregnancy outcomes associated in pregnant women who had received MQ. A large, multicentre RCT, which has evaluated the efficacy and safety of IPTp with MQ involving over 4,500 pregnant women has recently finished and is expected to provide further important information on MQ safety in pregnant women [65].

Only one article evaluating MQ safety in HIV-infected pregnant women [56] was found. However, the results of two small RCTs evaluating IPTp with MQ in HIV-infected women from Benin which reported similar results have been published recently [66].

Considering the overlapping geographical distribution of the HIV epidemic and malaria-endemic regions in sub-Saharan Africa, it is essential that studies on treatment and prevention of malaria in pregnancy include HIV-infected women and that research on potential drug interactions between anti-malarial drugs, including MQ, and antiretroviral drugs is undertaken [67].

## Conclusions

The use of MQ to prevent malaria in pregnancy should be based on a risk-benefit analysis that balances the likelihood of adverse effects against the risk of acquiring the infection, as is the case for other anti-malarials used in pregnancy. Women’s acceptability of a particular drug and their likely compliance also need to be considered when the choice of an anti-malarial drug for use in pregnancy is being considered. Based on the evidence reviewed, it can be concluded that MQ recipients did not have an increased risk of adverse pregnancy outcomes, including those in the first trimester of gestation.

There are only a few publications that have reported on maternal MQ tolerability in pregnant women when the drug has been used for malaria treatment, IPTp or prophylaxis. MQ combined with AS seems to be better tolerated than standard QN therapy for non-severe falciparum malaria but a MQ loading (10 mg/kg) dose is associated with more dizziness compared with placebo. When used for IPTp, MQ (15 mg/kg) may have more side effects than SP but this needs to be confirmed in double-blind randomized clinical trials.

There is a lack of RCTs evaluating MQ safety for malaria treatment and prevention in African pregnant women. Future trials should be designed to be double-blind to enable an objective assessment of MQ tolerability and need also to include HIV-infected women.

## Abbreviations

ANC: Antenatal clinic; AS: Artesunate; BMI: Body mass index; CDC: Centers for Disease Control and Prevention; CQ: Chloroquine; FDA: Food and Drug Administration; IPTp: Intermittent preventive treatment in pregnancy; ITNs: Insecticide treated nets; kg: kilogram; LBW: Low birth weight; mg: Milligram; MQ: Mefloquine; QN: Quinine; RCT: Randomized controlled trial; SP: Sulphadoxine-pyrimethamine; US: United States; WHO: World Health Organization.

## Competing interests

The authors declare that they have no competing interests.

## Authors’ contributions

All authors met International Committee of Medical Journal Editors’ criteria for authorship. RG and UH conducted the literature search and reviewed the articles included. BG and CM participated in the review design and methodology. RG wrote the draft manuscript. All authors revised the manuscript critically, and read and approved the final manuscript.

## Supplementary Material

Additional file 1 Table summarizing the studies that evaluated the safety of mefloquine for the prevention of malaria in pregnant women. Click here for file
