# Supplementary material for: Mefloquine safety and tolerability in pregnancy: a systematic literature review
Source: Malar J. 2014 Feb 28;13:75. doi: 10.1186/1475-2875-13-75 (PMC3942617; doi:10.1186/1475-2875-13-75)
Supplement: Additional file 1 — Table summarizing the studies that evaluated the safety of mefloquine for the prevention of malaria in pregnant women. [file 1475-2875-13-75-S1.docx]

**Additional File 1. Studies that evaluated the safety of mefloquine for the prevention of malaria in pregnant women**

| **Reference** | **Study year**  **and location** | **Study design** | **Study women** | **MQ safety on pregnancy outcomes** | **MQ tolerability** | **Comments** |
| --- | --- | --- | --- | --- | --- | --- |
| Nosten *et al.* 1990 | Thailand | Dose finding MQ pharmacokinetics study | N=20 women in 3^rd^ trimester  n=10 received 250 mg MQ/weekly  n=10 received 125 mg MQ/weekly | No data available | MQ was well tolerated in both MQ groups  Mild and short-lasting dizziness was reported in 7/10 women in each group | Small sample size  Open label |
| Balocco *et al.*1992 | 1990-92  Italy | Italian case series of 10 women exposed to MQ in 1^st^ trimester (data from Regional Drug Information Centre) | N=10 | No malformations or adverse pregnancy outcomes were observed | No data available | Limited data available |
| Nosten *et al.*  1994 | 1987-90  Thailand | RCT, double-blind, placebo controlled which evaluated MQ efficacy as prophylaxis  Phase 1: 500 mg MQ loading dose + 250 mg weekly for 4 weeks + 125 mg weekly until delivery *vs* placebo  Phase 2: 250 mg MQ weekly for 4 weeks and 125 mg weekly until term | Phase 1  N=60 MQ  N= 59 Placebo  Phase 2  N=111 MQ  N=109 Placebo | Phase 1:  Higher rates of stillbirths in the MQ group (7/56 *vs* 0/52)  Phase 2:  Similar rates between groups.  Overall (MQ *vs* Placebo):   - Abortions 1/159  *vs* 2/152 - Congenital anomalies: 4/159 *vs* 1/152 - Stillbirths 11/159 *vs* 4/152 | Phase 1:  28% dizziness in the MQ group *vs*  14% in the placebo group during 1^st^ week of prophylaxis  11% epigastric pain in MQ *vs* 27% in the placebo group  Phase 2:  Similar rates of reported adverse effects between MQ and placebo groups | Only RCT, double-blind and placebo-controlled trial evaluating MQ in pregnant women |
| Steketee *et al.*  1996 | 1987-90  Malawi | Trial, open, which compared 4 prophylaxis regimens:   1. CQ treatment + weekly prophylaxis 2. CQ treatment monthly 3. CQ weekly prophylaxis 4. MQ treatment + weekly prophylaxis | N= 4,187 enrolled, 3,793 analysed and divided as follows in 4 groups:   1. n=741 2. n=1459 3. n=661 4. n=932 (MQ) | Similar rates of abortions and stillbirths between groups  No congenital anomalies noted in either group. | The frequency of reported adverse effects was similar among the 4 study groups  MQ group was less likely to report itching and more likely to report dizziness than the CQ groups (exact numbers are not shown in the article)  One woman in the MQ group presented neuropsychiatric symptoms that resolved after drug discontinuation | Large trial  Open label  Limited information on MQ tolerability available |
| Smoak *et al.*  1997 | 1992-94  Somalia | Case series of US soldiers who inadvertently took MQ during pregnancy for prophylaxis. Information collected through questionnaires | N=72  (US soldiers) | No congenital anomalies were observed Among the 72 soldiers identified:   - 17 had an elective abortion - 12 spontaneous abortion (apparent increased risk) - one molar pregnancy - 23 live births - 19 unknown outcome | No data available | Small sample size  Recall bias possible |
| Vanhauwere *et al.*1998 | 1986-1996 | Analysis of the reports of exposure to MQ during pregnancy received by the Roche post-marketing surveillance system | N= 1,627 spontaneous reports of exposure to MQ in all trimesters | 4% of congenital anomalies in women exposed to MQ (similar rates to that found in the general population) | No data available | Data analysis of received reports (pharmacovigilance activities) |
| Philips-Howard *et al.*1998 | 1987-92 Europe | Analysis of reported exposure to MQ and other anti-malarials in 1^st^ trimester of pregnancy in a cohort of travellers and in a cohort identified from pharmaceutical data | Travellers:  N= 99 women exposed to MQ  N=118 exposed to CQ-proguanil  N=19 exposed to SP | Spontaneous abortion rates were higher in the MQ group (9.1%) than in the SP (2.6%), but comparable to background rates. Foetal anomalies were lower in the MQ group | No data available | Data analysis of received reports (pharmacovigilance activities) |
| Briand *et al.* 2009 | 2005-08  Benin | RCT open-label 2 dose IPTp-SP *vs* 2 dose IPTp-MQ | N=1,609  HIV negative | Incidences of miscarriages, stillbirths and congenital anomalies did not differ between groups (MQ *vs* SP):   - Miscarriages: 0.4 *vs* 0.1% - Stillbirth: 2.8 *vs* 2% - Congenital anomalies: 1 *vs* 0.5% | Proportion of reported adverse events (AEs) was significantly higher in the MQ group   - Vomiting: 54 *vs* 12% - Dizziness: 50 *vs* 13% - Tiredness: 40 *vs* 13%   AEs were more frequent at 1^st^ MQ intake than at second | Open label  First trial evaluating MQ for IPTp |
| Denoeud-Ndam *et al.* 2012 | 2005-08  (Trial 1) and  2009-12  (Trial 2)  Benin | Analysis that compared IPTp-MQ tolerability in HIV-infected and uninfected pregnant women, using safety data from cohorts of 2 different trials | N=385 HIV negative  (Trial 1)  N=94 HIV positive  (Trial 2) | Data not shown The frequency of stillbirths did not differ between MQ and the control group of each trial | Vomiting and dizziness were the most frequent reported AEs.  HIV-infected women reported less AEs than HIV negative   - Vomiting: 33 in HIV (+) *vs* 56% in HIV (-) - Dizziness: 39 *vs* 51% - Fatigue: 15 *vs* 42%   AEs were more frequent at 1^st^ MQ intake than at second. | Analysis comparing data from two clinical trials with different study procedures and among different populations (HIV-infected and uninfected women) |
| Schlagenhauf *et al.* 2012 | 1986-2010 | Analysis of the reports of exposure to MQ in pregnancy (all trimesters) received by the Roche post-marketing surveillance system | N=2,506 | Prevalence of birth defects (4.4%) and foetal loss is similar to background rates | No data available | Data analysis of received reports (pharmacovigilance activities) |
